# Corona mortis: clinical evaluation of prevalence, anatomy, and relevance in anterior approaches to the pelvis and acetabulum

**DOI:** 10.1007/s00590-023-03808-3

**Published:** 2024-01-10

**Authors:** Samuel Friedrich Schaible, Markus Simon Hanke, Christian Tinner, Johannes Dominik Bastian, Christoph Emanuel Albers, Marius Johann Baptist Keel

**Affiliations:** 1 Department of Orthopaedic Surgery and Traumatology, University Hospital Bern, Inselspital, University of Bern, CH-3010 Bern, Switzerland; 2 Trauma Center Hirslanden, Clinic Hirslanden Zurich, Medical School University of Zurich, Witellikerstrasse 40, CH-8032 Zurich, Switzerland

**Keywords:** Corona mortis, Pararectus, Modified Stoppa, Acetabulum, Pelvic Trauma, Anastomosis

## Abstract

**Purpose:**

To evaluate the clinical prevalence, characteristics, and relevance of the corona mortis (CM) in anterior approaches to the pelvis and acetabulum.

**Methods:**

Retrospective analysis of 185 theater reports from patients (73 females; mean age 62.8 ± 17.2 years) who underwent surgeries for pelvic ring injuries, acetabular fractures, or combined injuries using anterior approaches (Modified Stoppa or Pararectus) at our institution between 01/2008 to 12/2022. During procedures, the CM was routinely identified, evaluated, and occluded. Bilateral exposure of the superior pubic branch in 25 cases led to 210 hemipelvises analyzed. Exclusions: CM not mentioned in report and revisions via the initial approach.

**Results:**

In the 210 hemipelvises examined, the prevalence of any CM vessel was 81% (170/210). Venous anastomoses were found in 76% of hemipelvises (159/210), arterial in 22% (47/210). Sole venous anastomoses appeared in 59% (123/210), sole arterial in 5% (11/210). Both types coexisted in 17% (36/210), while 19% (40/210) had none. A single incidental CM injury occurred without significant bleeding. In ten cases, trauma had preoperatively ruptured the CM, but bleeding was readily managed. Females had a significantly higher CM prevalence than males (*p* = 0.001).

**Conclusion:**

Our findings show a CM prevalence aligning more with anatomical studies than prior intraoperative series. Although we observed one incidental and ten trauma-related CM injuries, we did not encounter uncontrollable bleeding. Our data suggest that in anterior pelvic approaches, when the CM is actively identified and occluded, it is not associated with bleeding events, despite its high prevalence.

## Introduction

**Fig. 1:**
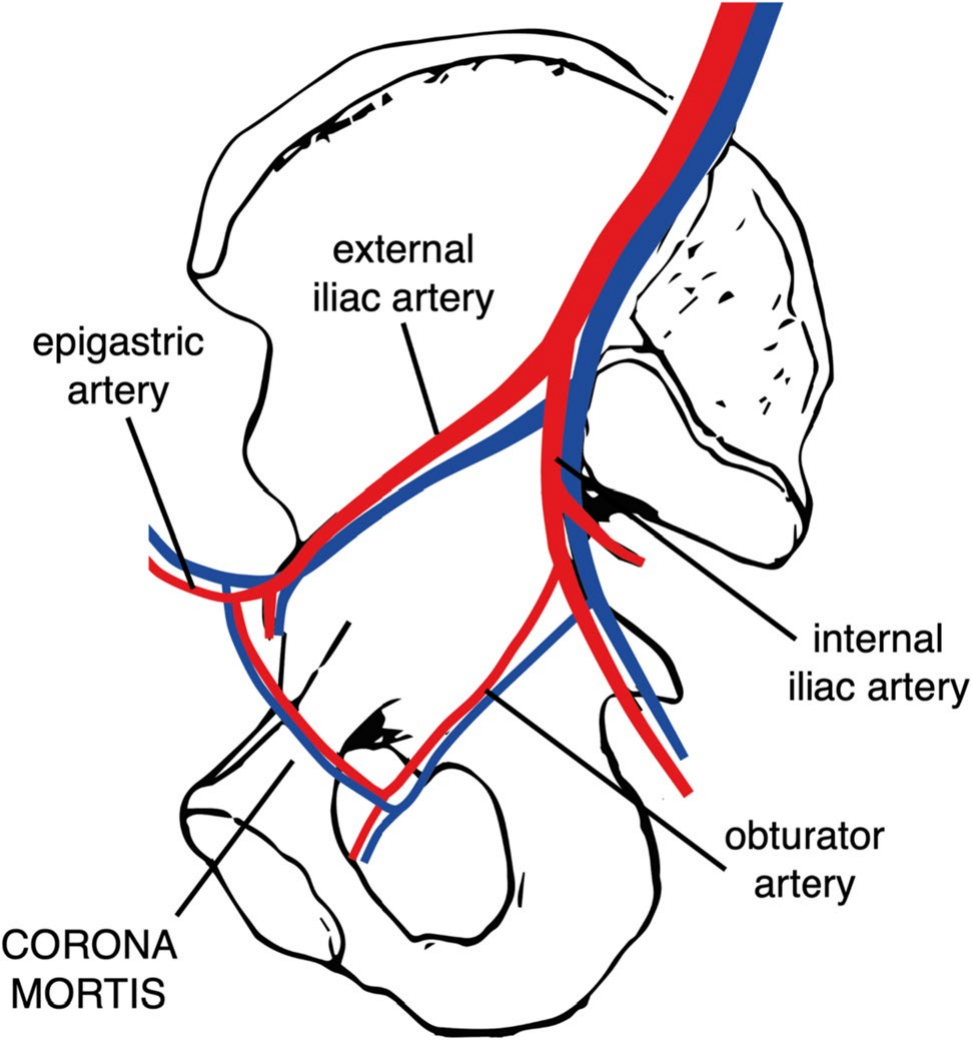
Schematic view of the CM with a medial view on the pelvis. *(Created in Adobe Illustrator)*

The corona mortis (CM), or “crown of death,” refers to arterial or venous anastomotic connections between the obturator and the external iliac or inferior epigastric vessels (Fig. 1). This term was introduced in the 34th edition of Gray’s Anatomy [1] and originates from clinical reports highlighting significant bleeding events associated with these vessels [2–6]. Letournel and Judet, among the first to use this nomenclature, questioned its clinical relevance, having encountered just one instance of this variant [7]. While anatomical studies indicate CM vessel prevalence of 33–83% [8–13], clinical studies report lower figures between 1–52% [3, 10, 14]. Only two studies in orthopedic pelvic surgery have addressed this topic, showing a prevalence of 1% and 38%. Both suggest minimal bleeding risk related to the variant [3, 10]. The primary aim of this clinical-anatomical study was to reevaluate the clinical prevalence of CM vessels in anterior approaches to the pelvis and acetabulum, considering the divergence between anatomical and clinical data and the sparse data on this variant in orthopedic surgery. Secondary goals included detailing its anatomy, assessing injury and bleeding risks, and comparing findings across genders—an aspect not previously explored.

## Methods

Following ethics approval from our institutional review board (KEK [Kantonale Ethikkommission Bern] No: Req-2022–00526), we retrospectively selected a cohort of 185 patients (73 females and 112 males) who underwent surgeries for pelvic ring injuries, acetabular fractures, or combined injuries using the Pararectus approach [15] or modified Stoppa approach [16]. All procedures were performed by the lead surgeon (MK) at our institution between January 2008 and May 2022. The mean age was 62.8 ± 17.2 (range: 14–94), and the mean BMI was 25.7 ± 5.0 (range: 18.0–33.4). In 25 cases, bilateral exposure of the upper pubic branch was performed, leading to the analysis of 210 hemipelvises. Theater reports were analyzed for references to the CM to assess its clinical prevalence, characteristics, and associated bleeding events. The surgeon qualitatively detailed the vessel’s morphology and estimated its diameter with millimeter precision. At our institution, we follow a standardized protocol for anterior pelvic approaches which involves the systematic identification, exposure, and occlusion of any CM found vessels. Small anastomoses with a diameter of less than five millimeters are coagulated, while those with a larger diameter are ligated or clipped. We only included reports by the lead surgeon that explicitly mentioned the presence or absence of the CM. Cases involving revisions via the initial approach were omitted due to potential anatomical alterations.

While comprehensively described elsewhere, briefly, the modified Stoppa approach starts with a Pfannenstiel incision above the symphysis, reaching both external inguinal rings. The linea alba is split longitudinally, potentially detaching the injured side’s rectus muscle, while maintaining an extraperitoneal approach and bladder protection. Blunt dissection along the pelvic brim exposes the CM vessels at the superior pubic branch. All anastomoses are coagulated, ligated, or clipped according to their size. The obturator and external iliac vessels are exposed, revealing the linea terminalis and the iliac bone’s medial portion. Detaching internal obturator muscle fibers medially to the sciatic spine fully exposes the quadrilateral surface [16]. In the Pararectus approach [15], an incision is made near the superior pubic ramus, along the rectus abdominis muscle’s lateral border. The externus aponeurosis and rectus sheath are divided, and the peritoneum is displaced medially. The retroperitoneal space is accessed, and the inferior epigastric vessels and the spermatic cord or round ligament are retracted. Here, the CM is identified and occluded as previously described. Elevation of the periosteum from the linea terminalis, lateral retraction of the iliacus muscle, and obturator muscle mobilization exposes the quadrilateral plate.

Modified Stoppa approaches were utilized in 75 patients (39 females, 36 males) for traumatic pelvic ring injuries, encompassing all Young–Burgess classification subtypes [17]. Lateral compression fractures were the most prevalent, primarily resulting from high-energy road accidents, falls, sports-related incidents, and low-energy traumas in osteoporotic individuals. The Pararectus approach was used in 110 patients (34 females, 76 males), mainly addressing acetabular fractures, or combined pelvic and acetabular injuries. Based on the Letournel classification [18], the predominant acetabular fracture types were anterior column, T-type, and two-column fractures. In the Pararectus group, low-energy traumas in osteoporotic patients were the most frequent cause, with other causes aligning with those in the Stoppa group. Patient demographics are detailed in Table 1.

**Table 1:** Patient demographics

Parameter	Value
Number of patients (number)	185
Female (number; [percent])	73 (39%)
Mean age (standard deviation)	62.8 (± 17.2)
Mean BMI (standard deviation)	25.6 (± 5.0)
Bilateral dissection (number)	25
Total Hemipelvises (number)	210
Acetabular fractures (number; [percent])	100 (54%)
Pelvic ring injuries (number; [percent])	63 (34%)
Combined acetabular and pelvic ring fractures (number; [percent])	22 (12%)

Statistical analyses and graphical representations were executed using RStudio for Mac Version 2023.03.0 + 386 (RStudio Team (2021). RStudio: Integrated Development for R. RStudio, PBC, Boston, MA URL http://www.rstudio.com/). The Shapiro–Wilk test was utilized to determine data distribution. Chi-squared tests compared the prevalence across groups. To compare CM diameters between groups, paired parametric student’s t tests were applied.

## Results

In all 185 theatre reports, there was consistent documentation of CM presence or absence. If present, the CM was described by its millimeter estimate, morphology, and management. At least one CM anastomosis, arterial and/or venous, was identified in 170 of the 210 hemipelvises analyzed, resulting in an overall prevalence of 81%. An arterial CM (with or without an accompanying venous CM) was observed in 47 hemipelvises (22%), and a venous CM (with or without an accompanying arterial CM) was found in 159 (76%). Isolated arterial CMs (without venous CM) were present in 11 hemipelvises (5%), while isolated venous CMs were observed in 123 (59%). In 36 hemipelvises (17%), both arterial and venous CMs coexisted. Of the 25 cases that underwent bilateral exposure of the upper pubic branch, 17 exhibited bilateral CMs (68%), with at least one CM vessel (arterial or venous) present on each side. Twenty-three hemipelvises (11%) displayed two or more venous anastomoses, and two had more than one arterial CM (1%). In these cases, two independent venous and arterial anastomoses were described. In two instances where the venous CM was characterized as ‘split and y-shaped,’ the anastomoses were counted as one. The CM was entirely absent in 40 hemipelvises (19%) (Table 2).

**Table 2:** Results overview

Parameter	Value
≥ 1 CM, any type (number; [percent^1^])	170 (81%)
Venous CM vein ± artery (number; [percent^1^])	159 (76%)
Arterial CM ± vein (number; [percent^1^])	47 (22%)
Only venous CM (number; [percent^1^])	123 (58%)
Only arterial CM (number; [percent^1^])	11 (6%)
Both CM types (number; [percent^1^])	36 (17%)
No CM (number; [percent^1^])	40 (19%)
Bilateral CM (number; [percent]^2^)	17 (68%)
Mean arterial diameter [mm]	3 (± 1)
Mean venous diameter [mm]	5 (± 1)

^1^of hemipelvises ^2^of cases with bilateral dissection

The mean arterial and venous CM diameters were 3 ± 1 mm (range: 2–6) and 5 ± 1 mm (range: 3–9). Seventeen venous anastomoses had a diameter above five millimeters, with one measuring just under a centimeter. Only one arterial anastomosis larger than five millimeters was found.

In ten of the 185 cases (5%), a venous CM was found to be ruptured and bleeding when identified during the surgical access. Eight of these cases were associated with high-energy trauma, and two with low-energy trauma. In eight of these cases, the bleeding was minimal and was managed through ligation. In two high-energy trauma cases, ruptured venous CM anastomoses displayed significant bleeding. In these cases, the bleeding was immediately controlled by oversewing the hole in the CM vessel using a 6–0 monofilament suture and clipping the obturator vein. In all other cases, the reported CM vessels were described as intact and patent and routinely occluded as previously described. In a singular case (0.5%), the CM was inadvertently injured by the surgeon during fracture mobilization, leading to notable bleeding from both venous and arterial CM anastomoses, despite earlier ligation during surgical access. The bleeding was effectively halted by clipping the obturator vein. No hemorrhagic events that caused hemodynamic instability, necessitating transfusion, or having a notable effect on the surgery were reported.

**Fig. 2:**
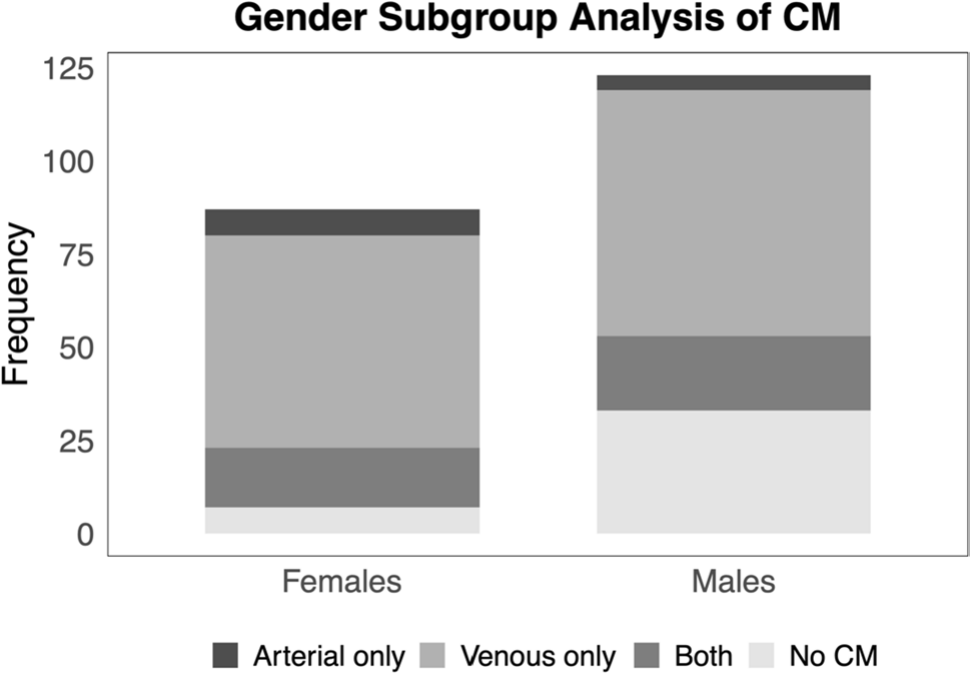
Number of hemipelvises with arterial, venous, arterial, and venous, or no CM (by gender). *(Created in R)*

A gender-based comparison revealed the presence of any CM anastomosis in 80 of the 87 female hemipelvises (91%) and in 90 of the 123 male hemipelvises (73%; *p* = 0.001). A venous CM (with or without arterial CM) was found in 73 female hemipelvises (84%) and 86 male hemipelvises (70%; *p* = 0.03). An arterial CM (with or without venous CM) was identified in 23 of the female hemipelvises (26%) and in 24 of the male hemipelvises (20%; *p* = 0.31). In females, both arterial and venous anastomoses were present in 16 hemipelvises (18%), while in males, this combination was found in 20 hemipelvises (16%; *p* = 0.59). The reported mean arterial diameter was 4 ± 1 mm (range: 2–6) in females and 3 ± 2 mm (range: 2–5) in males (*p* < 0.001). The mean venous diameter was 5 ± 1 mm (range: 3–9) for females and 4 ± 1 mm (range: 4–7) for males (*p* = 0.01). Gender comparison data are summarized in Table 3 and Fig. 2.

**Table 3:** Gender-based subgroup comparison

Group (Hemipelvises)	Female (87)	Male (123)	*p Value*
≥ 1 CM, any type (number; [percent])	80 (91%)	90 (73%)	*0.001*
Venous CM ± artery (number; [percent])	73 (84%)	86 (70%)	*0.03*
Arterial CM ± vein (number; [percent])	23 (26%)	24 (20%)	*0.31*
Both CM types (number; [percent])	16 (18%)	20 (16%)	*0.59*
Mean arterial diameter [mm]	4 (± 1.0)	3 (± 1.5)	< *0.001*
Mean venous diameter [mm]	5 (± 1.4)	4 (± 1.0)	*0.010*

## Discussion

The corona mortis (CM) is an arterial or venous anastomosis between the obturator and the external iliac or inferior epigastric vessels. When encountered in anterior pelvic approaches, it is associated with significant bleeding events [2–6]. Anatomical studies have observed a prevalence ranging from 33% to 83% [8–13]. Clinical series report a lower prevalence of 1–52% [3, 10, 14], and radiographic studies indicate a prevalence of 14–33% (arterial CM) and 29–51% (venous CM) [19–21] (Tables 4, 5, and 6). The aim of this study was to reevaluate the clinical prevalence, characteristics, and significance of the CM within the context of anterior approaches for osteosynthesis of the pelvic ring and acetabulum. Additionally, we aimed to compare findings across genders. Our methodology involved the retrospective analysis of 185 theater reports of anterior pelvic approaches performed by one surgeon, who systematically identified and managed the CM according to a standardized protocol. The data revealed an 81% overall prevalence for any type of CM (arterial or venous). The CM was more common in females than males. No significant bleeding events were reported in the entire cohort.

**Table 4:** Anatomic**/**Cadaver studies on the prevalence of corona mortis vessels

Author(s)	Year	Hemipelvises	Any CM (%)	Arterial CM (%)	Venous CM (%)
Tornetta P et al.	1996	50	42 (80%)	17 (34%)	35 (70%)
Berberoğlu M et al.	2001	14	n/a	n/a	12 (86%)
Sarikcioglu L et al.	2003	54	n/a	11 (20%)	8 (15%)
Okcu G et al.	2004	150	91 (61%)	29 (19%)	78 (52%)
Drewes P et al.	2005	30	10 (33%)	5 (17%)	9 (30%)
Damarnis S et al.	2007	80	66 (83%)	29 (36%)	48 (80%)
Pai M et al.	2009	98	n/a	2 (2%)	n/a
Rusu M et al.	2010	40	32 (80%)	26 (65%)	22 (55%)
Kacra B et al.	2011	10	n/a	n/a	4 (40%)
Nayak S et al.	2016	73	n/a	n/a	37 (51%)
Leite TL et al.	2017	60	n/a	27 (45%)	n/a
Chauhan MS et al.	2019	24	14 (58%)	2 (8%)	14 (58%)
Kati Y et al.	2021	12	10 (83%)	n/a	n/a

**Table 5:** Operative studies on the prevalence of corona mortis vessels

Author(s)	Year	Patients (Hemipelvises)	Approach	Any CM (%)	ACM^1^ (%)	VCM^2^ (%)	Summary/Conclusion
Teague DC et al.	1996	38 (38)	Ilioinguinal approach in acetabular fractures	14 (37%)	n/a	n/a	Prospective clinical study reporting a high variability of the retropubic vascular system
Berberoğlu M et al.	2001	28 (36)	Laparoscopic total extraperitoneal inguinal hernioplasty	n/a	31 (86%)	34 (94%)	Prospective study finding a higher venous compared to arterial CM prevalence
Lau H et al.	2003	121 (142)	Laparoscopic total extraperitoneal inguinal hernioplasty	56 (40%)	31 (22%)	38 (27%)	Attention should be paid to CM to reduce bleeding
Damarnis S et al.	2007	492 (492)	llioinguinal and modified Stoppa approach in pelvic ring injuries and acetabular fractures	5 (1%)	n/a	n/a	Retrospective concluding clinical CM prevalence is much lower compared to cadaver studies
Pellegrino A et al.	2014	25 (50)	Laparoscopic bilateral pelvic lymphadenectomy for gynecological oncologic procedures	26 (52%)	10 (20%)	18 (36%)	Prospective study finding higher venous CM prevalence followed by arterial CM and combined arterial and venous CM
Ates M et al.	2016	321 (391)	Laparoscopic total extraperitoneal inguinal hernioplasty	n/a	113 (28%)	n/a	Retrospective study concluding Cooper’s ligament should be stapled close to the symphysis to avoid CM bleeding
Selçuk I et al.	2018	96 (96)	Open pelvic lymphadenectomy in gynecologic oncologic patients	n/a	2 (2%)	n/a	Retrospective study questioning the relevance of CM vessels in gynecological surgery

^1^Arterial corona mortis ^2^Venous corona mortis

**Table 6:** Vascular radiographic studies on prevalence of corona mortis vessels

Author(s)	Year	Patients (HP^1^)	Procedure	Any CM (%)	ACM^2^ (%)	VCM^3^ (%)	Other Results
Serin E et al.	2002	98 (98)	Endovascular angiography in healthy individuals	n/a	n/a	28 (29%)	Arterial CM in 31% of males and 26% of females
Watkins GE et al.	2009	50 (100)	CT angiography in blunt pelvic trauma	18 (36%)	29 (29%)	n/a	Bilateral CM vessel in 11% of patients
Tang L et al.	2016	330 (660)	CT angiography in hospitalized women	n/a	93 (14%)	337 (51%)	Bilateral CM vessel in 43%, bilateral arterial CM in 6%, bilateral venous CM in 34% of patients
Rosen G et al.	2017	100 (200)	CT angiography in hospitalized patients	n/a	66 (33%)	n/a	Mean arterial CM caliber 2.3 mm
Montemezzi S et al.	2018	150 (300)	CT angiography in hospitalized patients	n/a	90 (30%)	n/a	Bilateral arterial CM in 19% of patients

^1^Hemipelvises ^2^Arterial corona mortis ^3^Venous corona mortis

The pronounced difference between the reported cadaveric and clinical prevalence has led to several theories to explain this discrepancy. Multiple authors suggest that the CM is susceptible to traumatic laceration either from direct mechanisms or indirect blunt trauma. Fracture displacement may sever these vessels upon injury, inducing vasospasm and complicating their intraoperative identification [3, 7, 9, 10]. Conversely, multiple clinical studies in non-orthopedic settings have reported lower incidences of the corona mortis, even in the absence of trauma, hence challenging this theory [14, 22, 23]. In this study, ten instances of a previously ruptured corona mortis occurred, eight from high-energy traumas and two from low-energy events, providing support for the trauma-induced laceration hypothesis. Nonetheless, despite all cases involving trauma, a patent CM was found in 81% of hemipelvises, and the injured vessels could still be identified. Further, the high prevalence of corona mortis in cadavers might originate from vessel occlusion, attributed to conditions such as arterial atherosclerosis in the elderly, promoting the formation of collateral circulation [24]. Moreover, it has been proposed that the variance in clinical observations arises from demographic and ethnic diversities in study samples [25]. Given the consistency of the finding across several studies with reasonably variable demographics that involved a variety of age groups, these theories seem tenuous. Another explanation could be rooted in the methodology and design of prior clinical investigations. Except for one [3], all identified clinical studies were retrospective. Some explicitly stated systematic identification of the CM in their methods [3], while others were vague [10]. As a result, assessments from these theatre reports might underestimate the CM’s true clinical prevalence due to potential oversight during surgery or inconsistent documentation. We excluded reports without mentions of the CM, minimizing potential oversight. In our study, by excluding reports without mentions of the CM, we minimized such oversight, leading us to conclude that the clinical prevalence of the CM closely aligns with anatomical studies. Furthermore, in the orthopedic context, the lower prevalence found by others might be related to the approach. In our experience, an intrapelvic approach facilitates identification of the CM. While our study exclusively employed intrapelvic approaches, other orthopedic series also incorporated extrapelvic approaches [3, 10]. Notably, a meta-analysis demonstrated decreased blood loss with intrapelvic approaches, potentially due to better recognition of critical anastomoses [26].

In this study, the most common variant observed was an isolated venous CM, found in 58% of hemipelvises, and the least common variant was an isolated arterial CM, found in 6%. This aligns with findings from anatomical cadaver research that differentiated between CM variants [8–10]. In line with these studies, a prevalence of 17% for hemipelvises with both venous and arterial connections was observed. Eleven percent of hemipelvises exhibited multiple venous CM anastomoses, while one percent presented multiple arterial CM anastomoses. These figures match the results from Darmanis et al., the only group to have reported such data in their cadaver study [10]. In 68% of the bilateral dissections, a CM of any kind was detected on both sides. This prevalence is marginally less than the 85% bilateral anastomoses reported by Darmanis et al.’s cadaver study [10]. Overall, the patterns of arterial and venous CM vessels were highly variable, as previously reported [3, 8–11]. Comparing CM vessel combinations with prior clinical research was challenging, as earlier studies typically reported aggregate CM prevalence without detailing specific configurations.

**Fig. 3:**
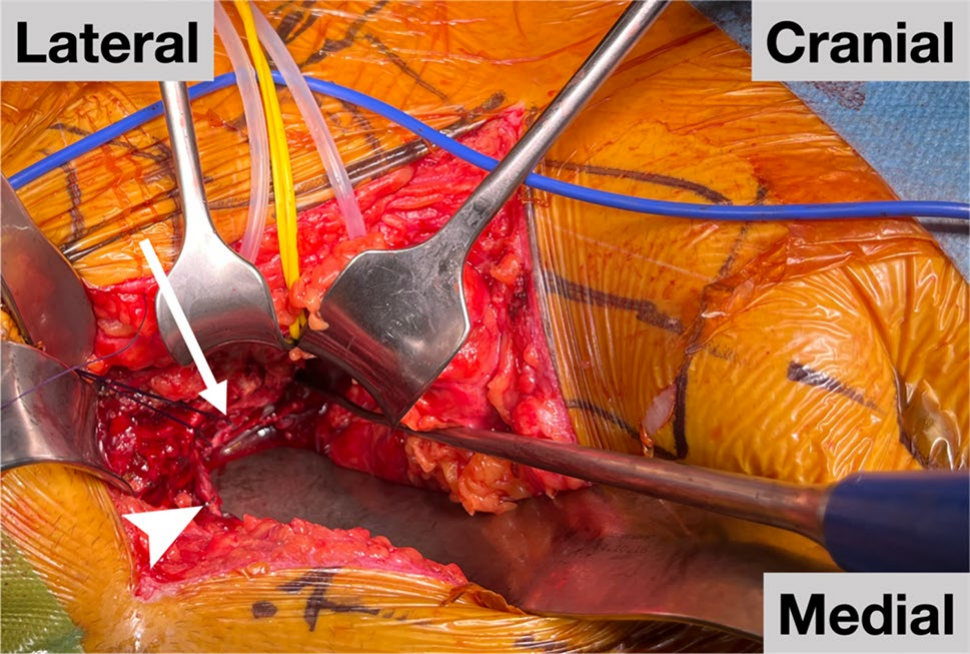
Intraoperative view of the corona mortis in a Pararectus approach. Arrowhead: Corona mortis (clipped). Arrow: Obturator vein located on the quadrilateral surface. *(Created in MS Powerpoint)*

Our study highlights a systematic approach to managing CM, with the surgeon actively identifying and occluding any CM vessels detected. One instance involved a CM injury due to fracture repositioning, even after previous ligation. Here, and in two cases of pre-ruptured venous CM, bleeding was halted by suturing the anastomosis and then clipping the obturator vein. Importantly, no such incidents, or any other, resulted in significant bleeding requiring intraoperative transfusion. Figure 3 illustrates the proximity of the CM and its connecting vessels to the site of acetabular fractures. Past research in both orthopedic surgery, and other disciplines has documented instances of considerable hemorrhage linked to inadvertent damage to CM variants [2–6, 10]. None of these studies advocated for actively identifying CM vessels. The proactive approach showcased here for anterior procedures aims to reduce hemorrhagic risks. Two authors recommended a similar strategy during laparoscopic total extraperitoneal hernia repair (TEP), but without endorsing vessel occlusion [22, 25]. In neither of the two studies routinely identifying the CM, incidental injury from the CM was reported. Together with our findings, this suggests that a proactive approach to the CM might decrease associated bleeding incidents. There are no reported adverse effects, such as abdominal wall necrosis, resulting from the occlusion of the obturator vein, the inferior epigastric vessels, or the CM itself [27], likely due to the formation of compensating collaterals. No complications associated with this step occurred in this series. In conclusion, actively identifying and occluding the CM during anterior approaches seems to be a safe strategy, possibly mitigating the risk of hemorrhagic complications associated with these vessels. Indeed, several publications focusing on anterior pelvic approaches (Pararectus and Stoppa) in orthopedic surgery endorse this approach [27–29].

In the gender subgroup comparison, females exhibited a higher prevalence of CM vessels. Several explanations could account for this: Females may inherently possess a higher anatomical occurrence of CM, or the CM could be more easily detected in females during surgeries, resulting in more frequent documentation. Females, compared to males, typically have a flatter pelvis, wider anterior iliac spines, and a narrower superior aperture, causing the artery to run more laterally to the symphysis pubis [30]. This could potentially enhance CM visibility. However, potentially confounding factors such as trauma mechanism and fracture type were not standardized between genders, which might skew the observed prevalence. Consequently, this study cannot establish a definitive relationship between gender and CM prevalence.

This study has both strengths and limitations. Its retrospective nature allows for extensive data collection but may introduce biases due to uncontrollable variables and potential confounders. The results heavily rely on the surgeon’s documentation, which, while systematic, remains subjective. A key strength is the study’s uniformity: It analyzes a consistent cohort of trauma cases, all operated on by one surgeon using two distinct techniques, ensuring meticulous attention to the CM. However, extrapolating these results broadly requires caution. While our findings are derived from a substantial sample size, rare events or nuanced implications of the CM might still be overlooked. While we effectively underscore the high clinical prevalence of the CM, further exploration is necessary to understand the implications of this observation, specifically the need for routine occlusion.

## Conclusion

This retrospective study underscores a substantial intraoperative prevalence of arterial, venous, and combined CM anastomoses. These rates are in alignment with cadaveric findings and surpass those from earlier clinical reports, suggesting that the cadaveric prevalence might accurately reflect real-world scenarios. Notably, our data indicate a higher prevalence of CM anastomoses in females. While the CM was frequently detected, there was a minimal bleeding incidence, with ten instances due to trauma and one iatrogenic injury. Routine identification and occlusion of the CM were consistently performed during procedures. While careful interpretation is warranted, our findings hint at the benefits of proactive CM management to mitigate bleeding risks, a concern often highlighted in literature.
